# Polysaccharides from Chinese herbs as natural weapons against colorectal cancer

**DOI:** 10.1042/BSR20230041

**Published:** 2023-05-18

**Authors:** Mingyue Zhou, Yinzi Yue, Yahui Wang, Shuai Yan

**Affiliations:** 1 State Key Laboratory of Quality Research in Chinese Medicine, Macau Institute for Applied Research in Medicine and Health, Guangdong-Hong Kong-Macao Joint Laboratory of Respiratory Infectious Disease, Macau University of Science and Technology, Macau, China; 2 Department of Anorectal Surgery, Suzhou TCM Hospital Affiliated to Nanjing University of Chinese Medicine, Suzhou 215009, Jiangsu, China

**Keywords:** cell apoptosis, colorectal cancer, inflammatory factors, intestinal microbes, Polysaccharides

## Abstract

Colorectal cancer (CRC) ranks third and second among the most widespread cancers worldwide and the most common causes of human death due to cancer, respectively. Furthermore, for unknown reasons, numbers of young patients diagnosed with colon cancer has increased. Polysaccharides are important functional phytochemicals reported to have anti-CRC effects. Moreover, CRC development and progression is closely related to the gut microbiome. Although approaches for treating CRC have been the subject of some review papers, research into traditional Chinese medicine (TCM) treatments for CRC and the underlying mechanisms involving polysaccharides have not been reviewed. Here, we reviewed the mechanisms underlying treatment of CRC using TCM polysaccharides, based on the etiology of CRC, and common treatment methods applied. The relationship between intestinal microbes and CRC, the mechanism by which TCM polysaccharides induce CRC cell apoptosis, and how TCM polysaccharides promote immune responses are discussed, as well as TCM polysaccharide use in combination with chemotherapy. TCM polysaccharides provide options for CRC treatment, due to their advantages of having multiple targets, eliciting modest adverse reactions, and wide range of available sources.

## Introduction

Colorectal cancer (CRC) ranks third and second among the most common cancers worldwide and the most widespread causes of human death due to cancer, respectively [1,2]. Recently, the incidence of CRC has been increasing, which is considered a global public health problem, due to changes in human diets [3]. CRC is caused by immortal cell proliferation due to accumulation of genetic and epigenetic alterations, and inflammatory cell infiltration among malignant and stromal cells [4]. In its early stages, CRC has no obvious symptoms, while bloody stool, local abdominal pain, and diarrhea may appear as the disease develops [5]. Therefore, the optimal time for therapeutic intervention has often passed at the time of diagnosis, which affects patient prognosis. The main clinical treatment methods for CRC include surgery, radiotherapy, chemotherapy, targeted therapy, and combination therapy; however, these approaches frequently cause complications, and long-term medication reduces patient compliance. Thus, developing novel and potent drugs for CRC treatment that exhibit less toxicity is an attractive goal.

Pharmacotherapy using natural substances is an emerging alternative to conventional treatments. Polysaccharides are crucial biomacromolecules with essential roles in the growth and development of living organisms [6]. Polysaccharides are polymer chains comprising ten or more monosaccharides with α or β glycosidic bonds generated by dehydration, are present in almost all living things, and are particularly abundant in traditional Chinese medicine (TCM). Furthermore, polysaccharides exert various pharmacological effects, including induction of tumor resistance, oxidation, effects on viruses and diabetes, protection from radiation, treatment of hypolipidemia, and immunomodulation, among others, with low toxicity and few side effects [7–20]. In TCM, polysaccharides are widely applied to treat CRC [21,22] and are reported to possess antiproliferative effects on colon cancer cells [23,24]. Moreover, polysaccharides can protect human normal cells and repair damaged DNA to restore their usual functions. Antioxidant and free radical-scavenging cells in the human body are prone to induce carcinogenesis under oxidative conditions [25]. Tumor-initiating factors induce cells to produce excess reactive oxygen species (ROS), which the cells cannot process, leading to oxidative damage of DNA molecules and mutation-driven carcinogenesis; however, polysaccharides can antagonize free radical peroxidation and improve antioxidant enzyme activity [26], and these highly beneficial effects of polysaccharides have been applied for cancer treatment. TCM has gradually become a hot spot of clinical research due to its excellent overall therapeutic effects, relatively few adverse reactions, and suitability for long-term use [27]. Traditional Chinese herbal polysaccharides provide additional possibilities for CRC treatment, due to their advantages of having multiple targets, inducing few adverse reactions, and being available from a wide range of sources.

The present review comprehensively summarizes naturally occurring polysaccharides with anticolon cancer effects and classifies the mechanisms underlying their multiple activities. The composition of the polysaccharides and their corresponding pharmacological effects are also explored. Published studies have demonstrated the importance of Chinese herbal polysaccharides applied for medical use, laying the foundation for further study, development, and application of these polysaccharides as functional foods and in modern medicine.

## Pathogenesis and treatment method of CRC

### Etiology and pathogenesis

**Figure 1:**
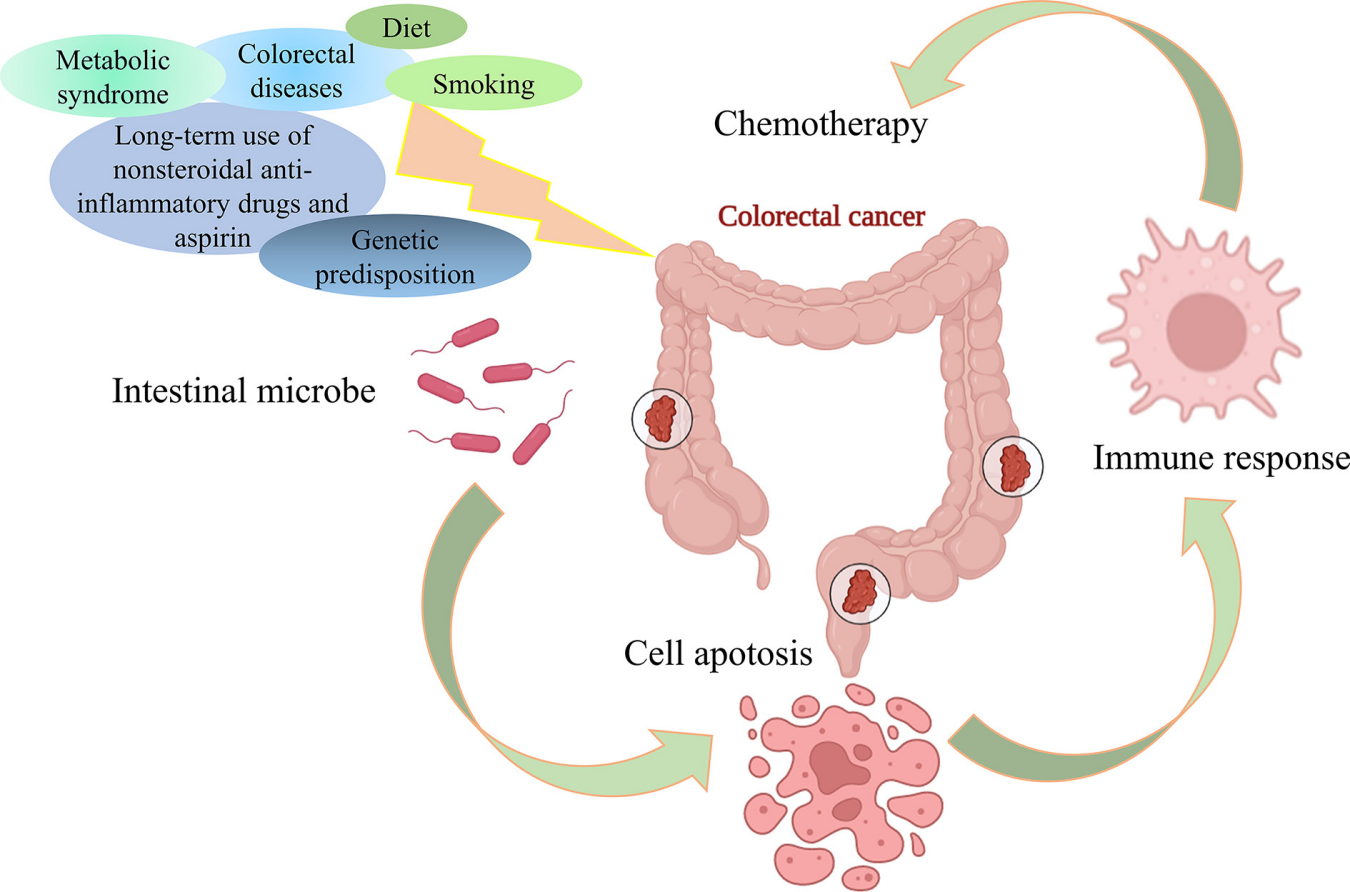
Schematic illustration of risk factors for CRC, including immunomodulatory mechanisms triggered by intestinal microbes

CRC is a relatively common cancer and has a high mortality rate [28]; however, the incidence rate has decreased with the development of modern medical treatments [29,30]. Changing patterns in CRC risk factors, earlier screening and diagnosis, and better treatment modes are considered reasons for improvements in CRC-related morbidity and mortality [31]. CRC can be diagnosed based on symptoms or screening. Early-stage CRC often has no symptoms or many that are nonspecific (e.g., changed bowel habits, abdominal discomfort, unexplained weight loss, persistent fatigue), and aggressive detection through a screening program is warranted (Figure 1) [32]. Diet has been identified as by far the most important exogenous factor influencing CRC etiology [33]. Red or processed meats, alcohol intake of ≥30 g per day, abdominal obesity, and factors that increase the height of adults can all contribute to CRC. Nondietary risk factors include smoking, long-term use of nonsteroidal anti-inflammatory drugs and aspirin, and specific conditions, such as some colorectal diseases, genetic predisposition, and metabolic syndrome [34]. Genetic susceptibility to CRC occurs in polyposis or nonpolyposis syndromes. The major polyposis syndrome is familial adenomatous polyposis (FAP), which is associated with mutation or loss of the FAP gene. Hereditary nonpolyposis CRC (HNPCC) syndrome is associated with germline mutations in six DNA mismatch repair genes. Adenomatous polyps are established precancerous lesions the occurrence of which, combined with the good survival associated with early-stage disease, makes CRC an ideal candidate for screening.

### Therapeutic method

To date, two screening strategies have been used for CRC: fecal occult blood testing and endoscopy. Symptoms of CRC include intermittent abdominal pain, nausea, or vomiting, secondary bleeding, obstruction, or perforation. The main treatment for nonmetastatic CRC is usually colon surgery, with chemotherapy as an adjuvant in some cases [35]; adjuvant therapies may benefit the survival of some patients with CRC after resection [36]. Results from randomized controlled studies suggest that adding oxaliplatin may benefit some patients; however, it is associated with significant toxicities, particularly chemotherapy-induced peripheral neuropathy [37–42]. Selection of adjuvant therapy in patients with CRC is based on consideration of disease stage and pathological characteristics, microsatellite instability, and the possible effects and toxicity of different treatments, as well as the age, comorbidities, and preference of the patient [43–46]. Surgery, chemotherapy, and radiation therapy have been the mainstays of CRC treatment; however, the use of TCM as a novel or add-on therapy for CRC is currently the subject of intensive study. TCM has been successfully used to relieve ileus in the perioperative period, and reduce ileus after operation and urinary retention after rectal operation [47,48]. Furthermore, the use of TCM polysaccharides, sometimes in combination with chemotherapy, has also had good results in patients with advanced CRC.

## Structure and anti-CRC activity of TCM polysaccharides

### Polysaccharide structure

Polysaccharides are important biopolymer compounds formed from numerous monosaccharides linked by bonds [49,50]. Polysaccharides and glycan are continuously synthesized and metabolized through the combined efforts of at least hundreds of proteins, and are derived from inorganic elements and small organic molecules, with no templates in living bodies. Moreover, as heterogeneous biomolecules, polysaccharides contain more structural information than protein, nucleic acid, and lipid complexes, leading to their vital functions in all organisms, including plants, animals, fungi, and microorganisms [51–56]. Polysaccharides have significant energy, structure, and biological functions in all living systems, and are the focus of increasing attention in the fields of biochemistry and medicine because of their curative effects and low toxicity [57,58]. Over 300 types of naturally occurring polysaccharide compounds have been identified to date [59].

Plants are important sources of natural polysaccharides, the functions of which are generally related to their structures, and include resistance to oxidation, tumors, and diabetes, immunomodulation, liver protection, hypoglycemia, and gastrointestinal protection [60–66]. As plants with medicinal value, Chinese herbal medicines are rich in polysaccharides, and data on the structures and functional activities of some of these polysaccharides have been generated. Most plant polysaccharides have been found to be relatively nontoxic and to cause no obvious side effects. Recently, due to the significant biological activity of Chinese herbal medicines, the polysaccharides extracted from them have become the focus of intense interest and some additional applications have been introduced, based on their structures and properties.

**Figure 2:**
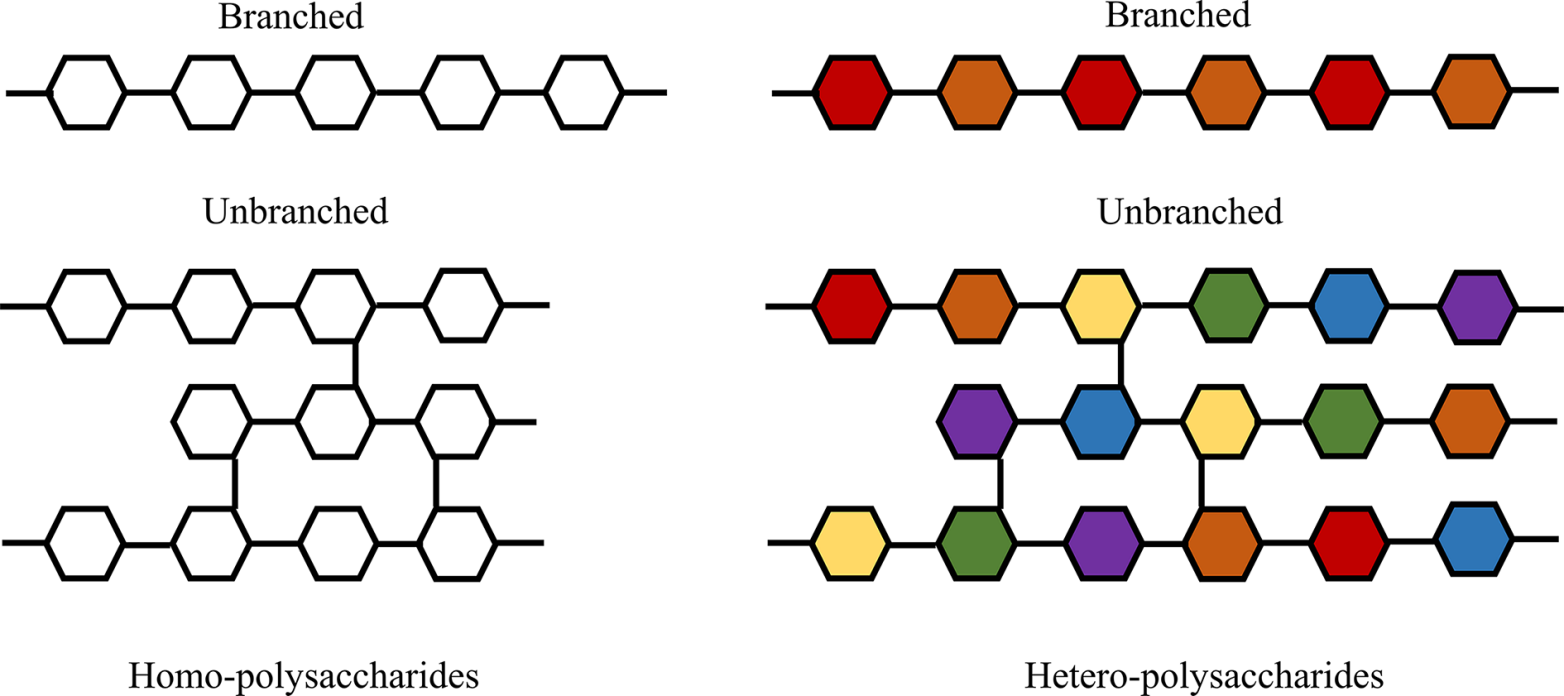
Diagrams illustrating homo-polysaccharide and hetero-polysaccharide structures; different colors represent different monosaccharides

Polysaccharide extraction methods, including the use of acid or enzyme-based extraction, microwave or ultrasound-assisted, and ultrahigh pressure-based approaches, have been developed, based on traditional aqueous extraction [10,67–74]. Polysaccharide separation and purification processes are performed after the removal of polysaccharides from aqueous extracts. Polysaccharides are commonly purified using gel chromatography, macroporous resin column chromatography, precipitation, anion exchange chromatography, and ultrafiltration, among other methods (Supplementary Table S1) [10,75–78]; however, due to difficulties in controlling polysaccharide quality, most of those obtained after extraction, separation, and purification remain crude products. Furthermore, different batches of the same herb may vary in terms of polysaccharide components, with small differences in terms of glycosidic bonds, molecular weight, biological function, and monosaccharide composition. Various bioactive polysaccharides from different Chinese herbal medicines have been purified, characterized, and reported, and research has mainly focused on their separation and purification, analysis of their monosaccharide composition, and determining their structures (including primary and higher structures, as well as structure–function relationships) [79–83]. Polysaccharides can consist of complex monosaccharide groups or chains comprising only a single monosaccharide, and have a broad molecular weight range (Figure 2), and can be classified into two types: homopolysaccharides and heteropolysaccharides. Polysaccharides comprising a single monosaccharide in a repeat chain are referred to as homopolysaccharides, while those composed of two or more monosaccharides are heteropolysaccharides. Homopolysaccharides may or may not contain branches, provided they contain the same monosaccharide units. The monosaccharide subunits of polysaccharides can be linked in a linear fashion or branched into complex structures. Moreover, monosaccharides containing one or more of sulfate, phosphate, or carboxyl groups to be considered acidic polysaccharides.

Polysaccharides have more complex structures than proteins and DNA, and this complexity makes analysis of their structures challenging. The rules of polysaccharide structural classification are the same as those for proteins and DNA, in that polysaccharide structures are classified in hierarchies from primary to quaternary [84]. Numerous analytical methods can be used to characterize the structures of polysaccharides, including chemical approaches, such as complete acid hydrolysis, partial acid hydrolysis, Smith degradation, and periodic acid oxidation; instrumental analysis methods, such as nuclear magnetic resonance spectroscopy (NMR), infrared mass spectrometry, and specific glycosidase digestion; as well as immune and other biological techniques [10].

The biological activities of polysaccharides are related to their structure, and particularly to their monosaccharide composition. Polysaccharides with α-helical structures have often been reported to have strong biological functions. Three different types of glycosidic linkages are primarily found in plant polysaccharides, including α-(1→6)-D, α-(1→4)-D, and β-(1→4)-D (Table 1). Linear polysaccharides (cellulose and hyaluronate) containing β-(1→4) glycosidic linkages and oxidized at C2 and C3 have a superior overall combination of structural features and can be used to improve drug delivery, including enhancing the efficacy of cisplatin against malignant tumors [85]. Furthermore, different types of polysaccharides exist within the same plant; for example, the polysaccharides, PAC-I, -II, and -III, with various molecular weights and differing monosaccharide compositions, are found in *Aloe vera* L. [86].

**Table 1:** Summary of structural and biological activities of polysaccharides used in TCM [10]

Glycans	Extraction methods	Major monosaccharides	Glycosidic linkage in backbone	MW (Da)	Bioactivities	References
*Astragalus membranaceus* polysaccharides	Hot water, ultrasonic and microwave extraction, DEAE-Sephadex A-25, Sephadex-G-100	Rhamnose, arabinose, xylose, ribose, galactose, glucose, mannose, fructose, fucose	α-(1→4)-Glc; α-(1→3)-Gal	8.7–4800 K	Antitumor	[89]
*Ginseng* polysaccharide	Hot water, ethanol fractionation, DEAE-Sepharose-CL-6B, Sepharose-CL-6B, Sephadex-G-75	L-arabinose, D-galactose, L-rhamnose, D-galacturonic acid, D-glucuronic acid	α-(1→3)-Ara; β-(1→3) or β-(1→4) Gal	3.2–1900 K	Antitumor	[90]
*Lycium Barbarum* polysaccharide	Warm water extraction, DEAE cellulose column, Sephadex-G-150	Glucose, arabinose, galactose, mannose, xylose, rhamnose, fucose, galacturonic acid, glucuronic acid	β-(1→3) or β-(1→4) Gal; α-(1→6)-Glc	Average 49.1 K	Antitumor	[91]
*Angelica* polysaccharide	Water extraction, Sephadex-G-100, DEAE-52	Glucose, mannose, galactose, rhamnose, arabinose, xylose	α(1,4)-Glc	5.1–2300 K	Antitumor	[92,93]
*Cordyceps sinensis* polysacchride	Hot water extraction, DEAE-Sepharose Fast Flow, Sephadex-G-75	Mannose, glucose, galactose, galacturonic acid	α(1→2) or α(1→4)-Man-α(1→4)-Glc	7.7–210 K	Antitumor	[94]

Various techniques are used to study and characterize the structures of polysaccharides, including high-performance liquid chromatography (HPLC), high-performance ion chromatography, UV–visible spectroscopy, gas chromatography (GC), Fourier transformed infrared spectra, X-ray photoelectron spectra, and NMR [87,88]. Moreover, extraction and separation methods, such as gel filtration chromatography (with Sepharose-CL-6B analysis), sedimentation, HPLC (using TSK-GEL columns), and high-pressure gel permeation chromatography, can affect the average molecular weight of polysaccharides (Supplementary Table S2).

### Activity of polysaccharides

Polysaccharide structures are closely associated with their biological activities, and their anti-CRC effects depend on their chemical composition and configuration [64,65,95]. Few studies have reported the relationships between polysaccharide structures and functions, and the links between the structures of polysaccharides and their anti-CRC activities are poorly defined; nevertheless, some relationships can be inferred. Sun et al. described a low-molecular-weight polysaccharide, SPS2p, whose monosaccharide composition is similar to that of *Ganoderma lucidum* polysaccharides (GLPs). Since the lower molecular weight of SPS2p allows greater physiological mobility of the polysaccharide, it shows a stronger anti-CRC effect than GLPs, which are easily bound by tumor cells [96,97]. The *β*-(1→6) linkage in the polysaccharide backbone is important for its anti-CRC activity, as it may enhance the effects of immunocompetent cells or induce tumor cell apoptosis [95,98,99]. The *β*-(1→3)-D-glucan obtained from *Lentinus edodes* is referred to as Lentinan and has potent antitumor and antiviral activities due to its unique properties [100]. Lentinan injection has been approved for cancer immunotherapy in Japan and is combined with chemotherapy in the clinic [101]. Furthermore, the bioactive D-fraction (*β-glucan*) of *Grifola frondosa* is undergoing clinical trials in the Japan and United States, due to its good antitumor activity [102]. The main polysaccharide present in the stem of *Dendrobium wardianum* (*D. wardianum*) is DWPP-Is, which has Mn and Mw values of 29.0 and 98.6 kDa, respectively [103]. The two monosaccharides most common in DWPP-Is are glucose and mannose, whose main chains include O-acetylated (1→4)-*β*-d-Manp and (1→4)-*β*-d-Glcp, which are structurally similar to other antitumor polysaccharides from *D. wardianum*. Ye et al. showed that treatment with DWPP-Is (10 mg per ml, in 0.2 ml) by gavage resulted in a similar rate of tumor inhibition as erlotinib hydrochloride (2 mg per ml) in A549 tumor-bearing KM mice. Therefore, DWPP-Is has potential as a functional drug to prevent lung cancer. Moreover, our understanding of the structural basis of the antitumor effects and underlying mechanisms of polysaccharides in this context has been deepened by a number of studies [60–62,64,65,95,104].

## Therapeutic mechanisms underlying the effects of TCM polysaccharides

Polysaccharides possess a wide range of biological functions. They can serve as a physiological energy supply and as the basic components of some substances, as well as participating in cell recognition, immune energy supply regulation, the transport of substances between cells, cell transformation, and tumor cell apoptosis. Among these functions, the antitumor activities of polysaccharides are the best studied [105,106]. Polysaccharides exert antitumor effects in two main ways: indirect inhibition or killing of tumor cells by improving immune energy supply, or direct antitumor effects, by inducing tumor cell differentiation or apoptosis and affecting oncogene expression [107,108]. Strengthening research into the antitumor activities of polysaccharides, as well as clarifying their antitumor mechanisms, will be important for the development of polysaccharides as antitumor or adjuvant therapeutic drugs.

### Polysaccharides influence CRC by regulating intestinal microbes

**Figure 3:**
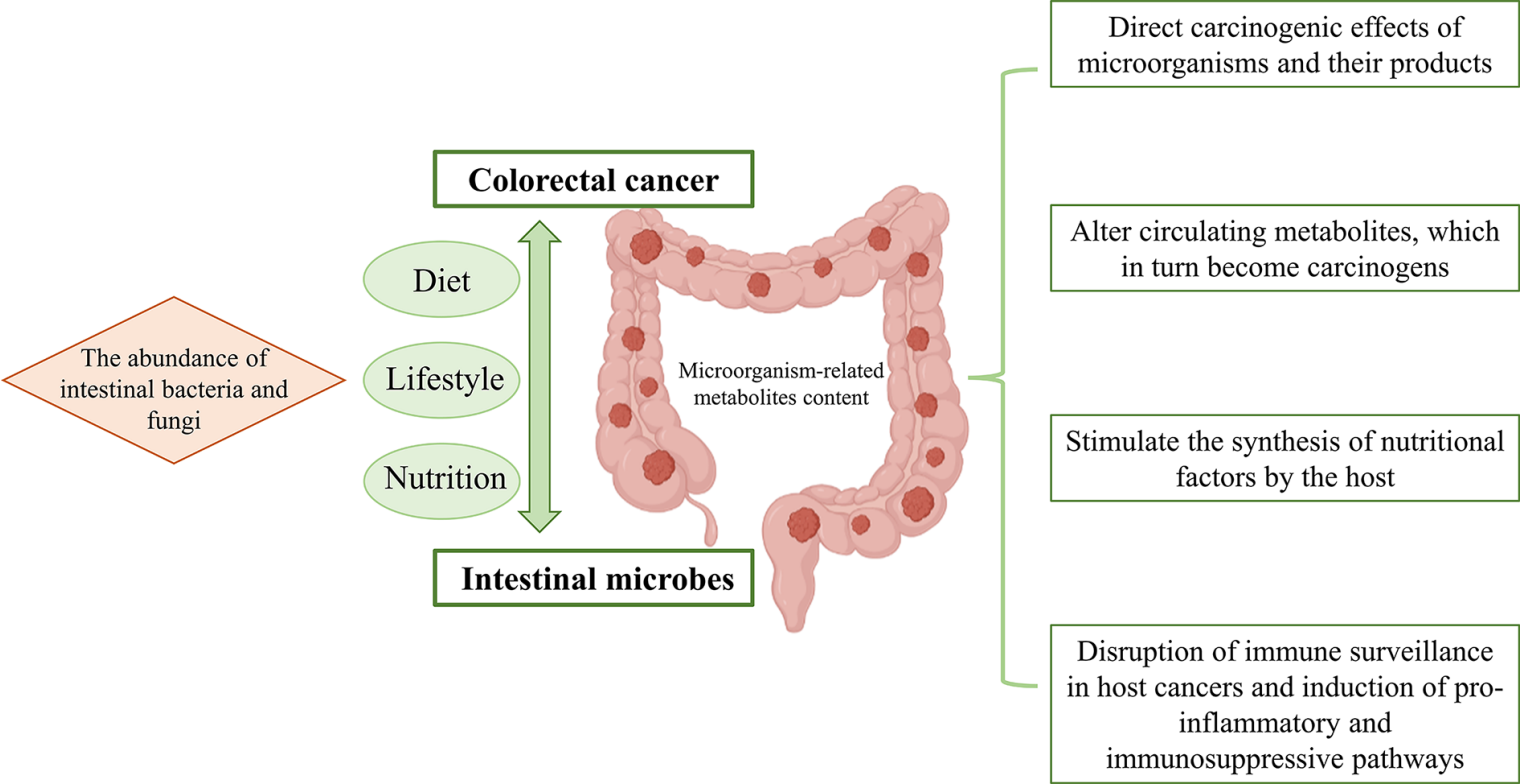
Schematic diagram of the relationship between intestinal microbes and CRC The microecological environment of intestinal microorganisms (left) induces somatic genetic changes and epigenetic changes in CRC. Four mechanisms by which intestinal microorganisms can promote tumorigenesis (right).

The colorectum has the largest microbial community in the body, and intestinal microecological and environmental factors can induce CRC-related somatic genetic and epigenetic changes; therefore, intestinal microbes must be considered in studies of CRC. Significant differences in the abundances of intestinal bacteria and fungi were observed between rectal cancer and sigmoid CRC, as well as in the content of various microorganism-associated metabolites [109]. The intestinal microbiota participate in CRC pathogenesis, and are a consideration in the diet, lifestyle, and nutrition factors that influence the development of the disease. Specific bacteria are related to CRC pathogenesis, as well as to the tumorigenesis pathways and molecular mechanisms that lead to CRC pathogenesis [110]. Currently, intestinal microbes are thought to promote tumorigenesis through four mechanisms: (1) direct carcinogenic effects of microorganisms and their products; (2) alteration of circulating metabolites, which in turn become carcinogens; (3) stimulation of the synthesis of nutritional factors by the host; and (4) disruption of immune surveillance in host cancers and induction of proinflammatory and immunosuppressive pathways (Figure 3) [111].

Polysaccharides contribute to various physiological activities, and intestinal microbes interact with polysaccharides in these contexts [112,113]. Polysaccharides serve as an important energy source for intestinal microbes and promote the proliferation of probiotics. Furthermore, intestinal microbial disturbances can lead to disease, and polysaccharides can ameliorate such conditions by modulating the intestinal microbiota. Ji et al. studied the effect of jujube polysaccharide (JP) on colitis tumors in mice induced by azomethane (AOM)/dextran sulfate sodium (DSS) [114]. JP treatment alleviated weight loss in mice and suppressed the number and size of colon tumors. High-throughput analysis of the intestinal microbiota of the mouse model demonstrated a significant increase in the relative abundance of Bacteroides, while that of Firmicutes decreased significantly, and the Firmicutes-Bacteroides ratio was decreased. Li et al. examined whether apple polysaccharides (AP) could prevent CRC by modulating intestinal microbiota disturbances [115]. AP treatment significantly decreased T cell and macrophages numbers, and suppressed WNT pathway activation, in mouse colon tissues. Moreover, numbers of Firmicutes were significantly reduced and those of Bacteroides significantly enhanced in the AP group, which restored the intestinal microbial abundances to the levels observed in control mice. Guo et al. studied the protective effect of *Nostoc commune Vaucher* polysaccharides (NVPS) on colon tumorigenesis in mice and the effects on intestinal microbiota [116]. Tumor number and size were significantly reduced after NVPS treatment. Furthermore, 16S rRNA gene sequencing and qPCR analysis of the bacterial composition in mouse fecal samples showed that NVPS significantly increased Firmicutes abundance and decreased that of Bacteroides, thereby altering the Bacteroides:Firmicutes ratio.

The role of polysaccharides in CRC prevention is mainly reflected in the reduction in tumor recurrence rates. Clinical trials have revealed multiple effects of polysaccharides in regulating intestinal flora, including promotion of intestinal probiotics, inhibition of harmful bacteria, improving the inflammatory environment, and reducing toxic carcinogen release, as well as effects on signaling pathways involved in adenoma formation. Moreover, short-chain fatty acids, which are metabolites of polysaccharide glycolysis by gut microbiota, suppress tumor cell proliferation and induce mucoprotein synthesis. This in turn strengthens tight junctions between epithelial cells, to prevent inflammation, and thereby inhibits intestinal tumor growth [117]. To date, research on polysaccharide regulation of intestinal flora and inhibition of CRC has mainly focused on the effect of polysaccharides in promoting probiotics; however, the specific mechanisms of action by which polysaccharides improve CRC by regulating intestinal flora require further study.

### Antitumor effects of polysaccharides

The antitumor effects of polysaccharides have been demonstrated by their significant inhibition of HeLa and lung cancer cell proliferation. Moreover, polysaccharides can inhibit the growth of xenografted sarcoma-180 tumors in mice [92]. The polysaccharide fractions, DNP-W1 and -W3, purified from *Dendrobium nobile* by Wang et al. showed strong inhibition of sarcoma-180 *in vivo* [118] and had better protective effects than other fractions against HL-60 cell growth. Furthermore, Jin et al. found that *Dendrobium candidum* polysaccharide (DP) inhibited the growth of mouse sarcoma-180 and enhanced thymus and spleen indices, as well as IL-2 and TNF-a levels, in mouse serum [119]. *In vitro* studies demonstrated a dose-dependent inhibitory effect of DP on human hepatoma SMMC27721 cell growth. Furthermore, DCPP-I-a and -II polysaccharides from *Dendrobium chrysotoxum* (*D. chrysotoxum*) significantly inhibited SPCA-1 cell proliferation, indicating that *D. chrysotoxum* polysaccharides are potential functional components that can contribute to cancer prevention [62].

Ginseng polysaccharides, including the acid polysaccharides, pectin, and arabinogalactan, consist mainly of L-arabinose, L-rhamnose, D-galactose, D-glucuronic acid, and D-galacturonic acid monosaccharides, with molecular weights in the range of 3.2–1900 kDa [120]. Ginseng polysaccharides have antitumor effects, inhibit cell proliferation, and induce HCT116 human colon cancer cell apoptosis via the cyclin inhibitor protein, p21 [121,122]. Furthermore, ginseng polysaccharides can significantly increase the level of endogenous antioxidants in the liver, without obvious hepatotoxicity [123], as well as restoring endogenous antioxidant enzymes, such as heme oxygenase-1, glutathione peroxidase, and superoxide dismutase, through their effects on cytokines [124,125].

*Angelica sinensis* (Oliv.) Diels (*A. sinensis*) polysaccharide can remarkably suppress HeLa and lung cancer cell proliferation and tumor growth in transplanted sarcoma-180 mice [92], associated with induction of tumor cell apoptosis by activating internal mitochondrial pathways, such as Bcl-2 family protein expression regulation, mitochondrial membrane disruption, increased levels of cytosolic cytochrome c, and improved caspase-9 and caspase-3 activities.

*Lycium barbarum* polysaccharides (LBP) include arabinose, glucose, rhamnose, fucose, xylose, mannose, and galactose [126]. The average-molecular weight of water-soluble polysaccharides from LBP was 49.1 kDa, and the molar ratio between arabinose and galactose was 5.6:1. Furthermore, LBP comprises highly branched polysaccharides, whose main chain consists of (1→6) Galp-linked galactose substituted at O-3 by galactosyl or arabinose [127]. LBP can inhibit the growth of various tumor cells, including liver cancer cells (SMMC-7721 and HepG2), cervical cancer cells (HeLa), gastric cancer cells (SGC-7901), and breast cancer cells (MCF-7); however, LBP does not inhibit the growth of normal hepatocytes. LBP suppresses cancer cell growth by inducing cell cycle arrest and apoptosis; LBP arrests the cell cycle in G0/G1 phase, alters mitochondrial function, activates oxidative stress, and regulates MAPK signaling to induce apoptosis [128].

**Figure 4:**
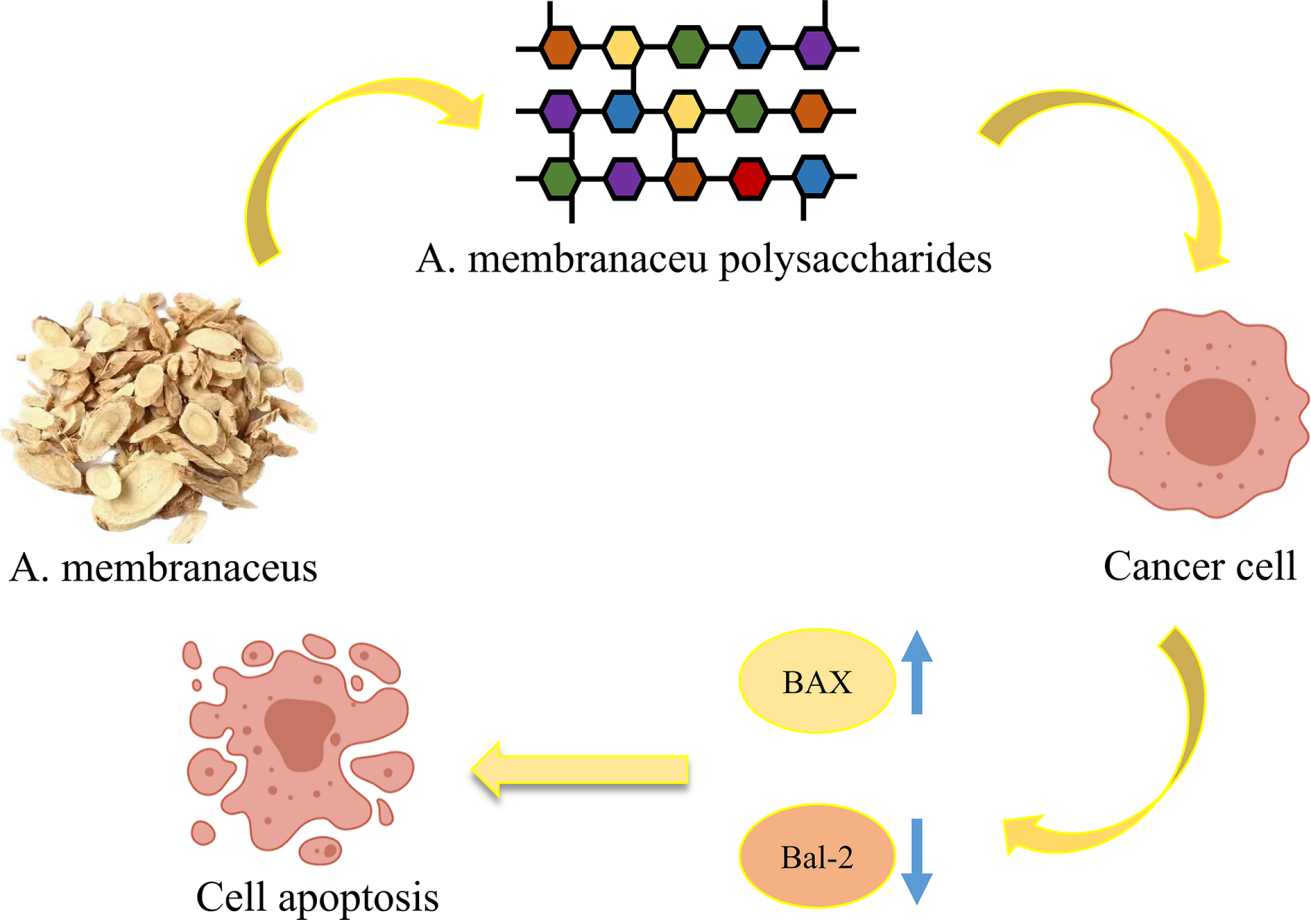
Summary of the mechanisms underlying the anticancer activity of *A. membranaceus* polysaccharides *A. membranaceus* polysaccharides promote HepG2 cell apoptosis by regulating the ratio of Bcl-2 and Bax.

*Astragalus membranaceus* (*A. membranaceus*) has immunomodulatory effects, and mediates resistance to inflammation, oxidation, glomerulonephritis, atherosclerosis, diabetes, and tumors. Jin et al. isolated 24 polysaccharides from *A. membranaceus* root, most of which were heteropolysaccharides (MW range, 8.7–4800 kDa) [89]. The proportions of monosaccharides isolated from Astragalus, including arabinose, glucose, fructose, fucose, mannose, galactose, xylose, ribose, and rhamnose, vary. Kiyohara et al. isolated 13 types of polysaccharide from the aerial parts of *A. membranaceus*, nine of which consisted of arabinogalactan and pectin, or arabinogalactan or pectin [129]. According to analysis of the structure of water-soluble *A. membranaceus* heteropolysaccharides, APSID3, the minimal repeat unit, includes one each of 1,5-linked arabinose, 1,3,4-linked rhamnose, 1,3-linked rhamnose, terminal arabinose; five 1,4-linked galacturonic acid residues; and six 1,4-linked glucuronic acids. The main chain includes 1,4-linked glucuronic acid, 1,4-linked galacturonic acid, and small number of attached 1,3-linked rhamnose, while the side chain comprises 1,5-linked arabinose on the C-4 of the 1,3-linked rhamnose [130]. Bcl-2 is an important factor involved in apoptosis that is primarily expressed in normal cell activation and development, but underexpressed or not expressed in apoptotic cells. Bax and Bcl-2 coregulate apoptosis; Bcl-2 is the most important apoptosis inhibitor, while Bax promotes apoptosis and forms a heterodimer with Bcl-2, and apoptosis depends on the ratio between the two [131]. *A. membranaceus* polysaccharides can inhibit Bcl-2 expression to induce tumor cell apoptosis [132]. Furthermore, *A. membranaceus* polysaccharides can promote HepG2 cell apoptosis by decreasing Bcl-2-induced hyperplasia and enhancing Caspase-3 activity (Figure 4) [133].

### Immune response promotion

**Figure 5:**
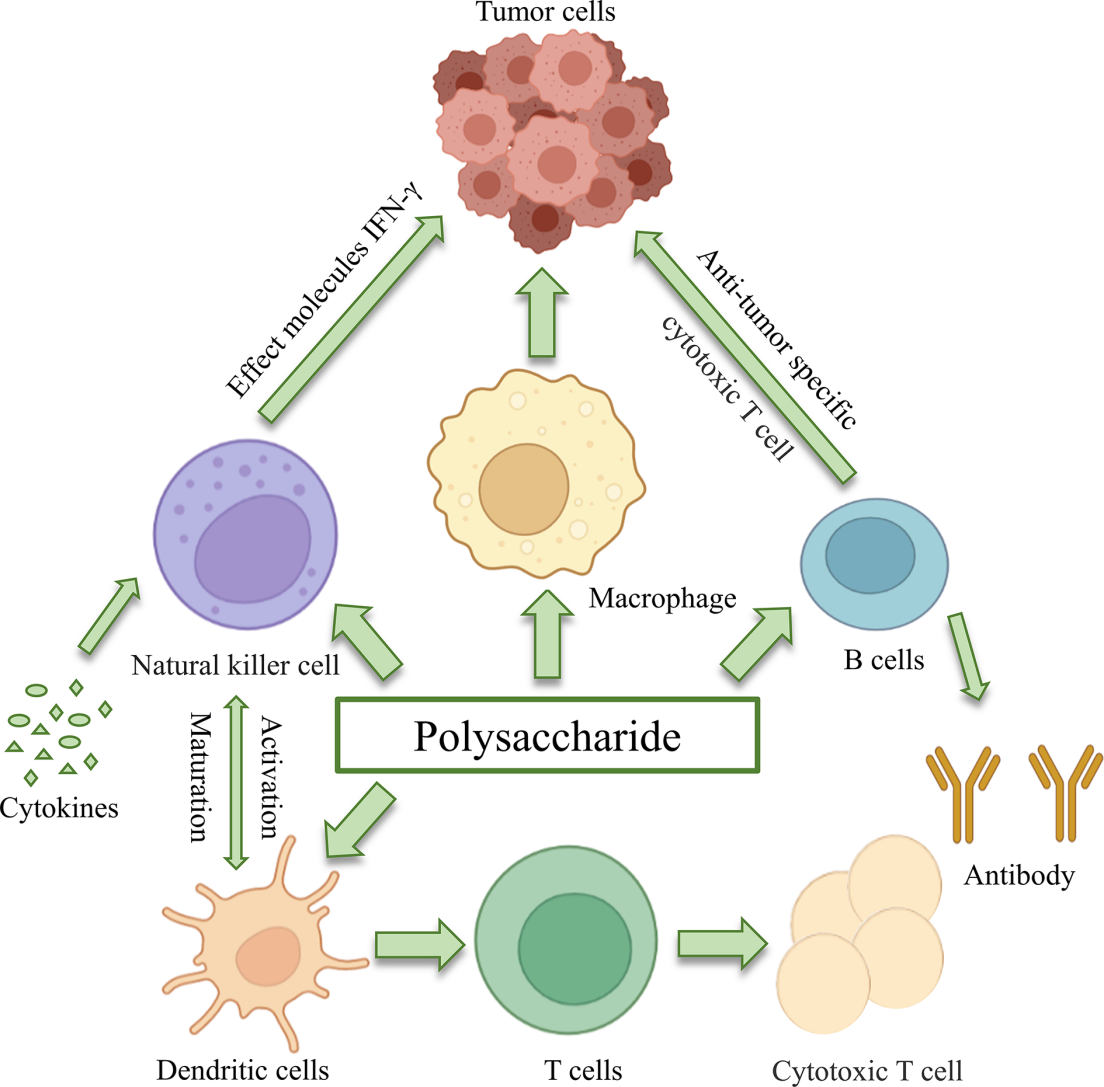
Immune regulatory mechanisms influenced by TCM polysaccharides

The immunomodulatory and antioxidant antitumor effects of polysaccharides have been extensively investigated, since immunosuppression and free radicals can directly induce tumor cell formation. The therapeutic effects of polysaccharides on tumors not only involve direct tumor cell killing but also involve enhancing immune defenses [134,135]. Polysaccharides have major effects in enhancing or activating macrophage immune responses, which include increased production of ROS, and enhanced cytokine and chemokine secretion (Figure 5).

Cao et al. found that the antitumor effects of polysaccharides may be due to macrophage and splenocyte activation, as well as stimulation of the secretion of some cytokines [136]. Yang et al. confirmed that polysaccharides can effectively inhibit solid tumor growth in BALB/c mice transplanted with H22 liver cancer, by reducing serum levels of IL-10, and promoting IL-12, IL-2, and TNF-a secretion [137]. Tian et al. also obtained similar results showing that polysaccharides can down-regulate MDR1 mRNA levels and P-glycoprotein expression in H22 tumor-bearing mice, as well as H22 liver tumor cells *in vitro* [138]. According to Sun et al., polysaccharides both significantly promote both cytokine production and gamma delta T-cell cytotoxicity, and enhances TNF-a and IFN-g levels, while reducing those of IL-10 and TGF-b in tumor-bearing mice [139].

Polysaccharides can also inhibit gastric cancer growth by stimulating an immune response [140]. Safflower polysaccharide (SPS) has antitumor and immune control effects, mainly through regulation of antitumor immune pathways. Wang et al. evaluated the antitumor activity of SPS on CRC. The active fraction SPS-1 isolated from SPS remarkably suppressed CRC induced by AOM/DSS and induced macrophage polarization to an M1 phenotype. Treatment with SPS-1 reduced the nuclear–cytoplasmic ratio, and caused regression of nuclear polarity and glandular hyperplasia, which alleviated the characteristic pathological symptoms induced by AOM/DSS [141]. SPS can also inhibit tumor growth by enhancing immunomodulatory activity and blocking the PI3K/Akt pathway [142].

A polysaccharide extracted from Lentinula edodes, referred to as lentinan, contains β-glucan, which has immunostimulatory effects, as the main biologically active component. Lentinan can activate immune cells through the TLR4/Dectin1-MAPK and Syk-PKC-NFκB pathways [143]. Many studies have shown that β-glucan is a very promising vaccine adjuvant, due to its stimulation of various immune responses, including antibody production, without any negative side effects [144]. *In vitro* studies have shown that β-glucan acts on several immune receptors, including the complement receptor, CR3, Dectin-1, and TLR-2/6, which also trigger the activities of macrophages, natural killer cells, monocytes, neutrophils, dendritic cells, and other immune cells [145].

*Inonotus obliquus polysaccharide* (IOP) is a major component of the parasitic *I. obliquus fungus*, and gavage with IOP reduces the weight loss, colon tissue damage, shortened colon, and proinflammatory mediator expression caused by colitis-associated cancer (CAC). Furthermore, IOP treatment enhanced NLRP3 inflammatory body expression, as well as IL-1β and -18 levels in the colons of CAC model mice. These results suggest that IOP inhibits the development of CAC, possibly by activating the NLRP3 inflammasome, and that IOP is a potential therapeutic candidate for CAC [146].

Li et al. found that *A. membranaceus* polysaccharide exhibits no direct cytotoxicity to 4T1 cells, while the effects on macrophages mediated by *A. membranaceus* polysaccharides can remarkably suppress 4T1 cell growth via inducing cell cycle arrest (G2 phase) and apoptosis [107]. *A. membranaceus* polysaccharide-induced macrophages mainly promote 4T1 cell apoptosis via the mitochondrial apoptotic pathway. Furthermore, *A. membranaceus* polysaccharide can remarkably increase the proliferation of mouse spleen lymphocytes and the phagocytic ability of peritoneal macrophages. Furthermore, *A. membranaceus* polysaccharide can up-regulate peripheral blood IFN-γ, TNF-α, and IL-2 expression levels, and together with 5-FU; *A. membranaceus* polysaccharide can enhance antitumor activity, where 5-FU is an immunosuppressive agent which can be used as an immune adjuvant for chemotherapy. *A. membranaceus* polysaccharides can also enhance the activity of dendritic cells, macrophages, natural killer cells, T and B lymphocytes, and microglia, as well as activating the expression of various cytokines and chemokines [147].

### Polysaccharide-combined chemotherapy

Cyclophosphamide (CTX) is often used in cancer chemotherapy, and high doses can cause immunosuppression and damage of the intestinal mucosa [148]. American ginseng (*Panax quinquefolius* L., AG) is effective in the treatment of immune disorders, colitis, and cancer [149,150]. Zhou et al. found that cotreatment with AG, particularly its polysaccharides and ginsenosides, can have an immunostimulatory effect, targeting the microbial metabonomics axis to prevent the side effects of CTX in patients with cancer [151]. Ouyang et al. demonstrated that GLPs can both improve the antitumor effects and reduce the side effects of chemotherapy in aggravating inflammation, oxidative damage, renal toxicity, and shortening survival [152]. The premise is that sufficient doses of GLPs are needed to be effective. There is evidence that oral (intestinal) glutamine can alleviate the symptoms and improve or maintain the life quality of patients with cancer. Supplementation with glutamine in a high-protein diet (10 g/day) plus disaccharides, such as sucrose and trehalose, can promote glutamine uptake by mucosal cells [153].

## Perspective

Polysaccharides in traditional Chinese herbal medicines are biologically active and have been the focus of extensive attention. It is of great interest to develop bioactive polysaccharides from traditional Chinese herbal medicines. Although mysteries remain in this field, the clinical value and prospects for wide application of plant polysaccharides make them an important direction in the development of new drugs. Many plant polysaccharides are applied as drugs or adjuvants to enhance the effects of drugs or decrease their adverse effects. With further study, the immune regulatory mechanisms involving plant active polysaccharides can be clarified, which will facilitate their further development and application.

Currently, the main focus of studies is polysaccharide extraction and purification, followed by analysis of their monosaccharide composition, and characterization of their structures and biological activities. Given the structural complexity and variety of types of polysaccharides, they are very challenging to isolate and purify, and structure determination is also difficult. Thus, the structures of most reported active polysaccharides remain poorly characterized. Many groups have conducted in-depth research into polysaccharides in Chinese herbal medicine, including their structural and biological properties. In recent years, analytical techniques, such as mass GC-MS, spectrometry, X-ray fiber diffraction, electron diffraction, and NMR have been used to determine structural information and mechanisms of action plant polysaccharides; however, many unresolved issues remain. One key issue is that the complex molecular structures of polysaccharides affect their degradation at the initial metabolic level and their absorption in the gastrointestinal tract.

The relationships between plant polysaccharide functions and their structures remain somewhat unclear. The activities of polysaccharides can vary widely, even among those from the same plant, demonstrating a direct relationship between polysaccharide structure and their biological activity [154]. Given the complex structures and various types of polysaccharides, their separation and purification, as well as structure determination, can be very difficult [155], and the characteristics and structures of most reported active polysaccharides are unknown. Therefore, effective new technologies to analyze the relationship between polysaccharide structure and their therapeutic effects, as well as their mechanisms of action in CRC, warrant further exploration. The focus should be on assessment of the relationships of polysaccharide biological activities with their chemical structures, which are closely associated, particularly their molecular weights, monosaccharide composition, and glycosidic bond types and positions [64,156,157]. Polysaccharides can have biological effects *in vitro* and in animal models; however, the cellular and molecular mechanisms have not been elucidated, and clinical research studies focusing on the treatment effects of polysaccharides and their food sources are needed.

The release of inflammatory mediators by polysaccharide-activated macrophages also warrants investigation. If excess inflammatory mediators are released, it may lead to excessive physiological inflammation [158]. Macrophages release a certain amount of nitrous oxide (NO) after polysaccharide treatment; the right amount of NO release can be expected to exert a beneficial effect and prevent adverse factors, while if macrophages are continuously stimulated, releasing too much NO, it can lead to sepsis, local or systemic inflammation-related disease, and even cancer. Key enzymes related to inflammation, such as iNOS and COX-2, have been implicated in cancers induced by long-term inflammation [159,160]. The use of plant polysaccharide-induced inflammatory factors in clinical applications, to maintain homeostasis but not cause damage due to excessive activation, should be studied. Furthermore, polysaccharides derived from both plants and microbes induce similar immune responses through binding to different receptors on the surface of macrophages, and macrophage responses to plant polysaccharides may mimic their innate immune response to microbial pathogens. Therefore, whether signals transmitted through different receptors are co-ordinated or interferential requires investigation. Furthermore, elimination of endotoxin pollution of active plant polysaccharides is necessary. Given the inhibitory effect of polymyxin B on lipopolysaccharide (LPS), polymyxin B is usually applied to indirectly detect LPS pollution; however, polymyxin B not only inhibits LPS activity but also affects iNOS expression and cytokine production to varying degrees [161]. Thus, controversy also exists about this test method, and development of safer and more effective tests to detect endotoxin contamination is another problem that requires an urgent solution.

Polysaccharides from TCM have unique monosaccharide compositions, structural diversity, and significant biological activity, and represent abundant natural resources for drug development. The advantages and disadvantages of polysaccharides lie in their complex molecular structures, and diverse biological functions and molecular targets. Therefore, it is necessary to further elucidate the structures of polysaccharides, their active constituents, and molecular targets related to drug activity. The pharmacodynamics, quality, and pharmacokinetics of polysaccharides also require extensive further study. These relatively cheap TCM polysaccharides could be used to develop new treatment agents, functional foods, or adjuvants, to prevent and treat various pathological conditions, in the same way as the fungal polysaccharides approved for application in China [55,162–165].

## Conclusions

CRC has a complicated pathogenesis, which is related to endocrine, immune, genetic, aging, nutritional status, exercise, external environment, and other factors. The complex pathogenesis process of CRC involves multiple systems, in which various signaling pathways exert important regulatory roles. The anti-CRC activity of polysaccharides has been confirmed both *in vitro* and *in vivo*, which provides strong evidence for their potential use to prevent and/or treat CRC. Multidimensional research on the etiopathogenesis of CRC and its current treatments, and the mechanisms of action of polysaccharides in CRC treatment (including the relationships between intestinal microbes and CRC, and between the mechanism of action of polysaccharides and CRC apoptosis induction, as well as that involved in polysaccharide promotion of physiological immune responses and polysaccharide MDT) has demonstrated the function and determine the mechanisms of action of TCM polysaccharides in CRC. TCM polysaccharides have the advantages of being multitargeting, multilayered, and multieffective, with minimal adverse reactions, and a wide range of sources, providing more possibilities for the treatment of CRC. Polysaccharides extracted from TCM have great potential for use in treatments and provide abundant sources for the discovery and development of new compounds for future medical application. The present review expands our understanding of these polysaccharides, laying the foundation for advanced study and development in this field.

## Supplementary Material

Supplementary Tables S1-S2

## Abbreviations

AG: American ginseng
AOM: azomethane
AP: apple polysaccharides
CAC: colitis-associated cancer
CRC: colorectal cancer
CTX: cyclophosphamide
DSS: dextran sulfate sodium
FAP: familial adenomatous polyposis
GC: gas chromatography
GLP: ganoderma lucidum polysaccharide
HNPCC: hereditary nonpolyposis CRC
HPLC: high-performance liquid chromatography
IOP: inonotus obliquus polysaccharide
JP: jujube polysaccharide
LBP: lycium barbarum polysaccharide
LPS: lipopolysaccharide
NMR: nuclear magnetic resonance
NO: nitrous oxide
NVPS: Nostoc commune Vaucher polysaccharide
ROS: reactive oxygen species
SPS: safflower polysaccharide
TCM: traditional Chinese medicine
TLR: toll-like receptors
