# Supplementary material for: Polysaccharides from Chinese herbs as natural weapons against colorectal cancer
Source: Biosci Rep. 2023 May 18;43(5):BSR20230041. doi: 10.1042/BSR20230041 (PMC10196152; doi:10.1042/BSR20230041)
Supplement: Supplementary Tables S1-S2 [file BSR-2023-0041_supp.pdf]

Supporting information

Research progress on the mechanism of polysaccharides from traditional Chinese herbs in the treatment of CRC

Table S1. The methods for extraction, separation, and purification of TCM polysaccharides.

Contents			Methods	Features
Isolation and purification	Miscellaneous	Remove protein	Sevage method	Troublesome, time-consuming, large amount of regents, structural damage, large losses
			Trichloroaceticacid	Effect but destroying structure
			Protease	Mild and efficient
		Decolorization	Actived carbon adsorption	Stronger affinity adsorption, large loss
			Hydrogen peroxide (H_2O_2)	Pigment containing unsaturated double bonds, hydroxyl groups, aromatic rings
			Ion exchange	High decolorization and retention rate
		Small molecule impurities	Dialysis	-
		Fractional purification	Precipitation	Polysaccharides with difference in solubility
			Gel chromatography	-
			Anion exchange chromatography	Crude purification of polysaccharides
			Microporous resin column chromatography	Have no effect on the biological activity
			Ultrafiltration	High separation efficiency, low energy consumption, no pollution and no damage to polysaccharide activity, easy to be contaminated

Table S2. Analytical method for identifying monosaccharide composition, molecular weight distribution, and glycosidic bonds of polysaccharides.

Items	Methods
Determination of purity and relative molecular weight distributions of polysaccharides	HPGPC, osmotic pressure, viscosity method, light scattering method, polyacrylamide gel electrophoresis
Monosaccharide compositional analysis	Complete acid hydrolysis, HPLC, GC, GC-MS, ion chromatography
Glycoside ring form (pyran, furan)	Infrared spectrum
Glycosidic linkages of the polysaccharide	Methylation analysis, GC-MS, LC-MS
The anomeric forms substituted by glycosides (α- and β-)	Glycosidase hydrolysis, nuclear magnetic resonance, infrared spectroscopy, laser Raman spectroscopy, etc.
Sequence of the oligosaccharides	Elective acid hydrolysis, sequential hydrolysis by glycosidases, nuclear magnetic resonance, etc.
The hydroxyl positions in the monosaccharide	Methylation, periodate oxidation, Smith degradation, GC-MS, nuclear magnetic resonance, etc.
Polysaccharide-peptide linkage	Dilute alkali hydrolysis method, hydrazine reaction, amino acid composition analysis, etc.
